# Antisense Oligonucleotide Induction of the hnRNPA1b Isoform Affects Pre-mRNA Splicing of *SMN2* in SMA Type I Fibroblasts

**DOI:** 10.3390/ijms23073937

**Published:** 2022-04-01

**Authors:** Jarichad Toosaranont, Sukanya Ruschadaariyachat, Warasinee Mujchariyakul, Jantarika Kumar Arora, Varodom Charoensawan, Bhoom Suktitipat, Thomas N. Palmer, Sue Fletcher, Steve D. Wilton, Chalermchai Mitrpant

**Affiliations:** 1 Department of Biochemistry, Faculty of Medicine, Siriraj Hospital, Mahidol University, Bangkok 10700, Thailand; jarichad.too@mahidol.edu (J.T.); sukanya.rus@mahidol.edu (S.R.); bhoom.suk@mahidol.edu (B.S.); 2 Department of Biochemistry, Faculty of Science, Mahidol University, Bangkok 10700, Thailand; warasinee.journal@gmail.com (W.M.); jantarikaarora@gmail.com (J.K.A.); varodom.cha@mahidol.edu (V.C.); 3 Integrative Computational BioScience (ICBS) Center, Mahidol University, Nakhon Pathom 73170, Thailand; 4 Systems Biology of Diseases Research Unit, Faculty of Science, Mahidol University, Bangkok 10400, Thailand; 5 Perron Institute for Neurological and Translational Science, The University of Western Australia, Perth, WA 6009, Australia; norman.palmer@perron.uwa.edu.au (T.N.P.); s.fletcher@murdoch.edu.au (S.F.); s.wilton@murdoch.edu.au (S.D.W.); 6 Centre for Molecular Medicine and Innovative Therapeutics, Health Futures Institute, Murdoch University, Perth, WA 6150, Australia

**Keywords:** spinal muscular atrophy, hnRNPA1, antisense oligonucleotide, phosphorodiamidate morpholino oligomer, transcriptome

## Abstract

Spinal muscular atrophy (SMA) is a severe, debilitating neuromuscular condition characterised by loss of motor neurons and progressive muscle wasting. SMA is caused by a loss of expression of *SMN1* that encodes the survival motor neuron (SMN) protein necessary for the survival of motor neurons. Restoration of SMN expression through increased inclusion of *SMN2* exon 7 is known to ameliorate symptoms in SMA patients. As a consequence, regulation of pre-mRNA splicing of *SMN2* could provide a potential molecular therapy for SMA. In this study, we explored if splice switching antisense oligonucleotides could redirect the splicing repressor hnRNPA1 to the hnRNPA1b isoform and restore SMN expression in fibroblasts from a type I SMA patient. Antisense oligonucleotides (AOs) were designed to promote exon 7b retention in the mature mRNA and induce the hnRNPA1b isoform. RT-PCR and western blot analysis were used to assess and monitor the efficiency of different AO combinations. A combination of AOs targeting multiple silencing motifs in hnRNPA1 pre-mRNA led to robust hnRNPA1b induction, which, in turn, significantly increased expression of full-length SMN (FL-SMN) protein. A combination of PMOs targeting the same motifs also strongly induced hnRNPA1b isoform, but surprisingly SMN2 exon 5 skipping was detected, and the PMO cocktail did not lead to a significant increase in expression of FL-SMN protein. We further performed RNA sequencing to assess the genome-wide effects of hnRNPA1b induction. Some 3244 genes were differentially expressed between the hnRNPA1b-induced and untreated SMA fibroblasts, which are functionally enriched in cell cycle and chromosome segregation processes. RT-PCR analysis demonstrated that expression of the master regulator of these enrichment pathways, MYBL2 and FOXM1B, were reduced in response to PMO treatment. These findings suggested that induction of hnRNPA1b can promote SMN protein expression, but not at sufficient levels to be clinically relevant.

## 1. Introductions

Spinal muscular atrophy (SMA), a severe, debilitating neuromuscular condition, is the leading genetic cause of death in children. SMA is caused by insufficient expression of survival motor neuron (SMN) protein, most commonly stemming from genomic deletions of the entire *SMN1* gene. The complete absence of the SMN protein is incompatible with life, as established by the unviability of *Smn*-knockout models. The SMA condition manifests in humans due to the presence of a near identical gene, *SMN2*, that could potentially encode a normal SMN but which is inappropriate for splicing of the majority of *SMN2* transcripts, and hence, low expression of the full-length SMN (FL-SMN). The aberrant splicing of *SMN2* pre-mRNA leads mainly to mature *SMN2* mRNA transcripts missing exon 7 that are translated into a nonfunctional protein. hnRNPA1 was identified as a crucial splice repressor regulating inclusion of exon 7 in the *SMN2* transcript [1,2,3,4]. Depletion of the hnRNPA1 expression was shown to promote inclusion of *SMN2* exon 7 into mature transcript, and therefore, an increase in SMN protein [5,6]. Antisense oligonucleotides (AOs), synthetic short single-stranded nucleotide with chemical modifications on the sugar backbone of AOs, were designed to target the binding site of this repressor (ISS-N1) [2]. Treatment of this AO efficiently increased SMN protein in cultured fibroblast from a patient with SMA [7] and animal models [8,9]. This compound (Spinraza^®^) is now commercially available for SMA patients. The development of this treatment has highlighted the fact that AO mediated splice switching is a viable molecular treatment option for patients with SMA [8,10]. The class of the AO backbone to produce Spinraza^®^ is the methoxyethyl modified AO chemistry; this class of backbone chemistry was previously associated with proinflammatory effect, thrombocythemia or kidney complications in some of the clinical trials [11,12,13]. Herein, we propose the use of AO to alter the splice repressor hnRNPA1 to another isoform as a means to induce inclusion of *SMN2* exon 7 into mature transcript, in case Spinraza^®^ causes major adverse effects later on.

hnRNPA1 encodes two main isoforms: hnRNPA1 (320 amino acid) and hnRNPA1b (372 amino acid). The hnRNPA1 isoform is predominantly expressed in most tissues (GTEx portal). The hnRNPA1b transcript is an alternatively spliced isoform with the inclusion of exon 7b encoding a protein with 52 additional amino acids in a glycine/arginine rich region, a domain that is reportedly important in protein–protein interactions [14]. Interestingly, the overexpression of hnRNPA1b is reported to yield an alternative 5′ splice site selection and overexpression of hnRNPA1 protein, as determined by in vitro splicing assay [15].

This study investigates whether AO-mediated shifting of the hnRNPA1 isoforms towards hnRNPA1b expression can alter the splicing pattern of *SMN2* and augment SMN protein expression. We employed an RNA sequencing approach to determine the effects and consequences of this hnRNPA1 isoform shifting on the transcriptome in SMA patient fibroblasts.

## 2. Results

### 2.1. Individual AO Induction of hnRNPA1 Exon 7b Inclusion

**Figure 1:**
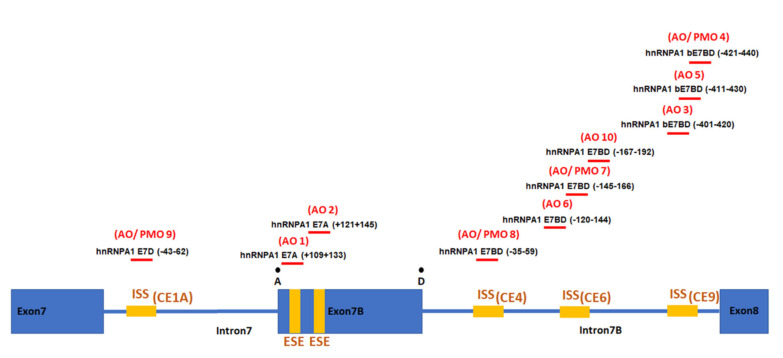
Schematic diagram displaying the targeting splice motifs and AO coordinates of ten AOs induced hnRNPA1 exon 7b skipping (AOs1 and 2) and inclusion (AOs3–10).

**Figure 2:**
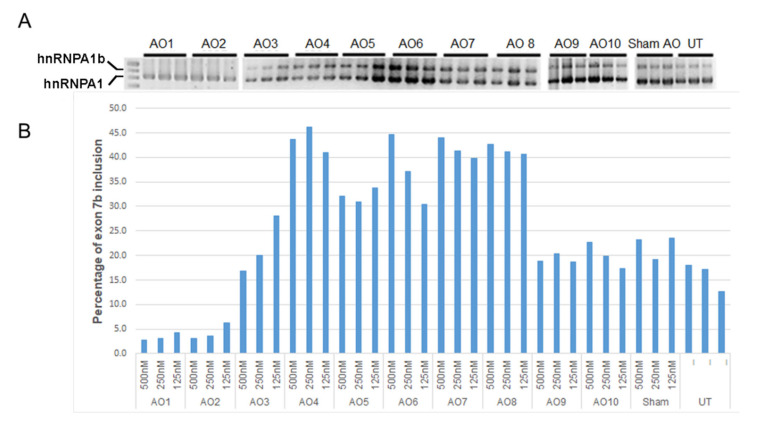
RT-PCR analysis demonstrated splicing patterns of hnRNPA1, (**A**) and percentage of exon 7b inclusion in SMA fibroblasts transfected with different 2′O-methyl modified AOs (**B**).

In this study, ten 2′ O-methyl modified AO candidates were evaluated for their effectiveness in modifying the hnRNPA1 splicing pattern. The panel of ten AOs was designed to target either the predicted ESE motif (AOs 1 and 2) or previously reported ISSs (AOs 3–10) (Figure 1) [16]. The splicing pattern of hnRNPA1 transcripts was determined by RT-PCR analysis, with a typical gel image displayed in the upper panel of Figure 2. A densitometric analysis of hnRNPA1 isoforms was performed, and is displayed as mean percentages of hnRNPA1 exon 7b inclusion (Figure 2B). AOs 1 and 2 were designed to mask predicted exonic splice enhancers (ESE) and, as expected, this led to substantial skipping of exon 7b with reduced overall amount of hnRNPA1 transcript. Three AOs (AOs 4, 7 and 8) in particular promoted an approximately 40% increase in hnRNPA1 exon 7b inclusion at all tested concentrations.

### 2.2. Systematic Assessment of Different AO Combinations Targeting Multiple Silencing Motifs to Induce hnRNPA1b Exon 7b Inclusion

We explored whether combinations of AOs could improve the efficiency of hnRNPA1b induction, as “looping out” had been proposed as a mechanism for hnRNPA1 exon 7b skipping [15]. This mechanism indicates that hnRNPA1 protein binds to splicing motifs located in adjacent introns of hnRNPA1 exon 7b to induce exon 7b skipping. This led us to focus on using AOs to sterically and simultaneously block these conserved elements (CE) by using combinations of AOs. One AO, AO9 targets a motif in intron 7, ISS(CE1A), whereas AOs 8, 7 and 4, targeting the other three ISS motifs, i.e., ISS(CE4), ISS(CE6) or ISS(CE9) respectively, were chosen based upon the efficiency of single AO treatment (Figure 1). We then combined AO9, that masks ISS(CE1A), with AOs targeting the other three motifs (viz. ISS(CE4), ISS(CE6) or ISS(CE9)), giving a total of seven possible combinations (see Table 1).

**Figure 3:**
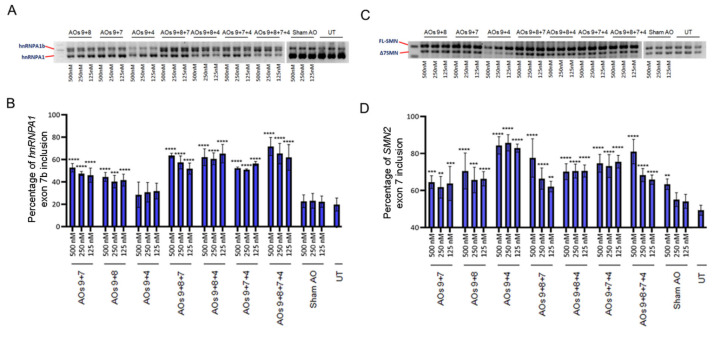
Gel images and densitometric analysis of RT-PCR of hnRNPA1 (**A**,**B**) and SMN (**C**,**D**) splicing isoforms in SMA fibroblasts transfected by seven combinations of AO cocktails, as well as densitometric analysis of the percentage of FL-SMN. The indicated concentrations were a dose of combined AOs in the cocktail. The * symbol indicates the *p*-value of the average percentage of exon inclusion compared to untreated samples (viz ** = *p* < 0.01, *** = *p* < 0.001 and **** = *p* < 0.0001).

These combinations were then tested at three different concentrations, i.e., 500 nM, 250 nM or 125 nM, in three separate experiments, and a RT-PCR analysis of hnRNPA1 and SMN splice isoforms was undertaken to test the efficiency of the various AO cocktails (Figure 3). The AOs9 + 8 + 7, AOs9 + 8 + 4 and AOs9 + 8 + 7 + 4 combination yielded approximately 10–15% more of the hnRNPA1b transcript compared to the two AO cocktails targeting a similar motif (viz. AOs9 + 8 or AOs9 + 7). Moreover, increased levels of the hnRNPA1b transcript with higher concentrations of AOs9 + 8 + 7 correlated with changes in inclusion of *SMN2* exon 7. AOs9 + 4 led to decreased overall amount of hnRNPA1 transcripts with substantially increased inclusion of *SMN2* exon 7, despite only 5–10% of hnRNPA1 exon 7b being induced (Figure 3).

### 2.3. AO Combinations Targeting Multiple Silencing Motifs as a Means of Inducing hnRNPA1b Protein

**Figure 4:**
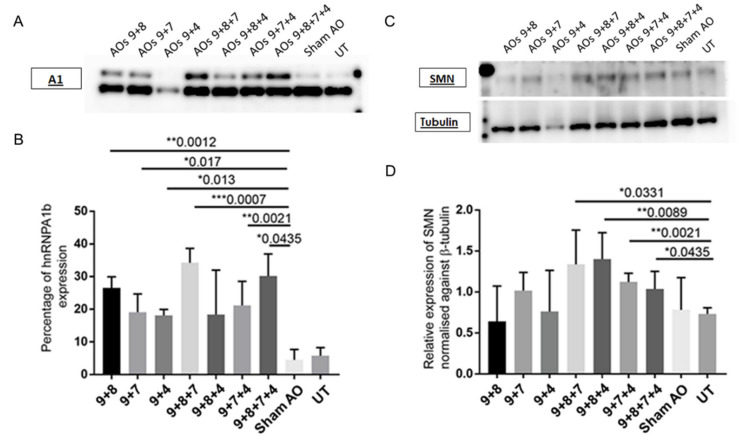
Western blot analysis indicates the hnRNPA1 (34 kDa) and the induced isoform of hnRNPA1 proteins at approximately 38 kDa (**A**), SMN protein at approximately 38 kDa (**C**) and β-tubulin protein at approximately 50 kDa (**C**) expressed in SMA fibroblasts that were transfected with selected seven different AO cocktails, as well as densitometric analysis of percentage of hnRNPA1b (**B**), as compared to SMN protein expression normalised against β-tubulin (**D**). The * symbol indicates the *p*-value of the average relative protein expression compared to untreated samples (viz * = *p* < 0.05, ** = *p* < 0.01 and *** = *p* < 0.001).

Transfection with each AO cocktail at a concentration of 500 nM was examined to establish whether induced transcript levels are associated with increased protein expression. Most combinations significantly increased hnRNPA1b expression with slight increase in the overall amount of hnRNPA1 transcript. Levels of induction ranged from 20% to 35%, compared to 5% of hnRNPA1b expression in untreated and sham transfected fibroblasts. The AOs9 + 8 + 7 cocktail also significantly increased FL-SMN expression in SMA fibroblasts compared to sham treated control (Figure 4).

**Figure 5:**
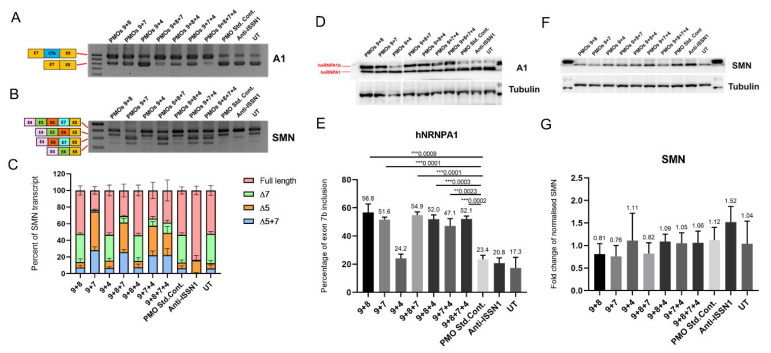
Gel images of RT-PCR shows hnRNPA1 (827 bp) and hnRNPA1b (983 bp) transcripts (**A**). Panel (**B**),(**C**) shows gel images of different isoforms of *SMN2* transcripts missing exon 5 (Δ5), missing exon 7 (Δ7) or missing both exons 5 and 7 (Δ5 + 7) with densitometric analysis of *SMN2* transcript. Western blot analysis of hnRNPA1, FL-SMN and β-tubulin expression in SMA fibroblasts transfected with selected seven different PMO cocktails as well as densitometric analysis of percentage of hnRNPA1b compared to SMN protein expression normalised against β-tubulin (**D**–**G**). Numbers above each bar graph indicates the average values of percentage of hnRNPA1b isoform (**E**) and the average fold change of increased SMN protein (**G**). ** = *p* < 0.01, *** = *p* < 0.001.

We synthesised AOs 9, 4, 7, and 8 as phosphorodiamidate morpholino oligomers to further enhance efficiency of exon skipping, and the different backbone chemistries showed greater biological stability. Most combinations, except for PMOs9 + 4, strongly induced 50 to 60% of hnRNPA1b protein in a cultured SMA fibroblast. PMO cocktails that contain the PMO 7 in the mixture, i.e., PMOs9 + 7, PMOs9 + 8 + 7, PMOs9 + 7 + 4, and PMOs9 + 8 + 7 + 4, caused skipping of *SMN2* exon 5 (Figure 5C). None of the PMO cocktails evaluated was found to augment FL-SMN protein expression (Figure 5).

### 2.4. Transcriptomic Analysis of PMOs 9 + 7 + 8 Induced hnRNPA1b Isoform in SMA Fibroblasts

**Figure 6:**
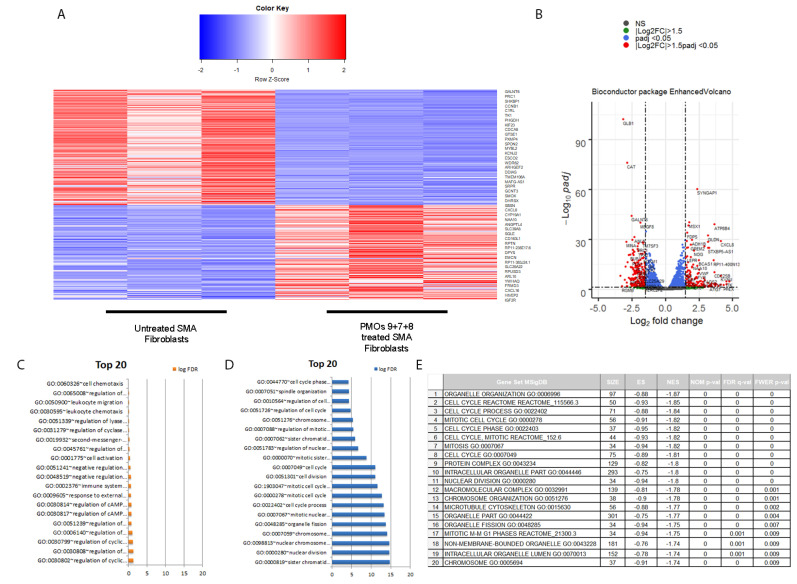
Differential expression comparing between PMOs9 + 7 + 8 treated SMA fibroblast and untreated SMA fibroblasts. Heatmap of the differentially expressed genes in three replicates of both conditions. (**A**) Volcano plot of differentially expressed gene list comparing between cells transfected with PMOs9 + 8 + 7 and untreated SMA fibroblasts. (**B**) Selected genes with absolute log2 fold change greater than 1.5 folds and *p*-value lesser than 0.05 was analysed for its gene ontology. Top twenty of biological processes that were upregulated (**C**) or downregulated (**D**) in PMO treatment group compared to untreated control. Gene set enrichment analysis indicated that gene set regulating cell cycles were enriched at the normalised enrichment score (NES) of −1.8477 and false discovery rate (FDR) q-value of 0.00 (**E**).

**Figure 7:**
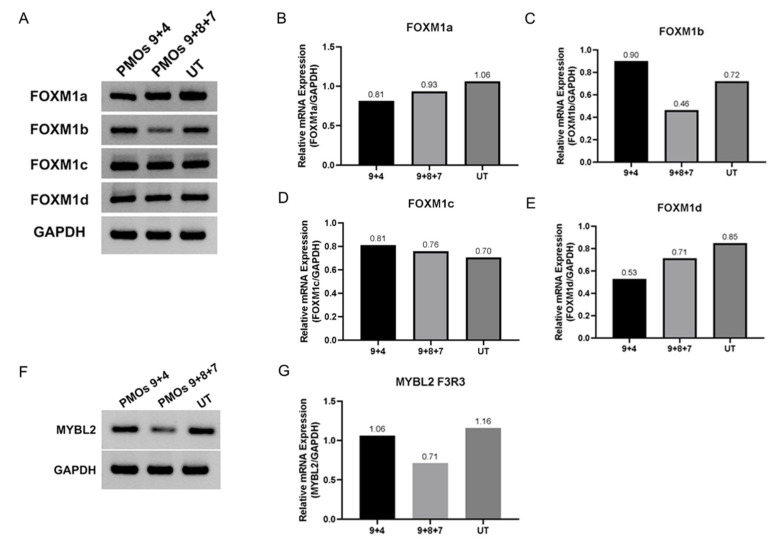
Isoform specific RT-PCR to detect four different isoforms of FOXM1 (**A**) with the graph displaying band densitometry in the panels (**B**–**E**) (viz FOXM1a, FOXM1b, FOXM1c and FOXM1d) showed reduced expression of FOXM1b in cultured SMA fibroblasts treated with the combination of PMOs 9 + 8 + 7, while RT-PCR analysis of MYBL2 (**F**) was also reduced, as indicated in its band densitometry (**G**).

We further investigated the effect of PMOs9 + 7 + 8 induced hnRNPA1b expression at the transcriptomics level in cultured SMA fibroblast. A panel of differentially expressed genes between the combined PMOs9 + 7 + 8 treated and untreated SMA fibroblasts was detected and is displayed as a volcano plot in Figure 6A. A panel of differentially expressed 3244 genes were identified, as the absolute log2 fold change was greater than 1.5 with *p*-value lesser than 0.05. Expression of Survivin (BIRC5), Aurora kinase B (AURKB) and marker of proliferation ki-67 (MKI67) were approximately four-fold less in the PMOs9 + 7 + 8 treated SMA fibroblasts compared to untreated fibroblast with the adjusted *p*-value of 1.52 × 10^−24^, 3.58 × 10^−6^ or 2.91 × 10^−6^, respectively (Supplementary Table S3). A gene ontology analysis of these genes indicated that these differentially expressed genes involved the cycle control, mitosis, nuclear division and chromosome segregation (Figure 6B,C). We further performed a gene set enrichment analysis (GSEA) on this gene set and found that these genes were enriched in the cell cycle process, with a normalised enrichment score of −1.8477 and a FDR q-value of 0.00 (Figure 6D). FOXM1 and MYBL2 are strong candidates for regulating the cell cycle, nuclear division and chromosome segregation controlling protein, as either FOXM1 or MYBL2 was present in nine out of twenty enriched pathways identified by the GSEA analysis. FOXM1 has four isoforms, i.e., FOXM1A, FOXM1B, FOXM1C and FOXM1D, and the isoform specific RT-PCR indicated that only FOXM1B was reduced by transfection with PMOs9 + 7 + 8, but not with PMOs 9 + 4 or in untreated SMA fibroblasts, while MYBL2 was also reduced in SMA fibroblasts treated with this PMO cocktail (Figure 7).

## 3. Discussion

Regulation of *SMN2* exon 7 inclusion has become a most promising focus as a molecular therapy for SMA patients. Despite potentially encoding an identical SMN protein, the predominant *SMN2* gene transcripts in SMA are missing exon 7 and encode a defective unstable protein. Shifting the *SMN2* splicing pattern towards the normal SMN full-length product has been proven to be effective in increasing SMN protein levels with concomitant improvements in phenotype [2]. Different strategies (viz. small molecules and AOs) have also been utilised to modify the process of *SMN2* exon 7 inclusion [9,17,18,19].

SF2/ASF (SRSF1) and hnRNPA1 are two crucial splicing factors that are responsible for control of differential splicing of the *SMN1* and *SMN2* gene transcripts. SF2/ASF binds to an ESE located in *SMN1* exon 7, [5,20] while a C>T polymorphism at the sixth nucleotide of *SMN2* exon 7 creates an ESS that is recognised by hnRNPA1, thus leading to omission of exon 7 from the mature *SMN2* mRNA [3,4]. In addition, one silencer element located in *SMN2* intron 7 (ISS-N1) that has been used as a target for FL-SMN induction exerts its *SMN2* exon 7 skipping effect through its binding to hnRNPA1 [3,4].

As a splice repressor, hnRNPA1 binds to pre-mRNA through two RNA recognition motifs (RRMs) located in the amino terminal region of hnRNPA1 protein, [16] and the glycine/arginine rich carboxyl terminal region is reported to be critical in protein–protein interactions and pre-mRNA splicing [15]. hnRNPA1b contains an extra 52 amino acids inserted into the glycine/arginine rich carboxyl terminal region, and overexpression of hnRNPA1b is known to lead to alternative 5′ splice site selection rather than overexpression of the full-length isoform [21].

Eight AOs targeting four silencing motifs [16] were designed and tested individually for their effectiveness in inducing hnRNPA1b exon 7b inclusion. Transfection of SMA fibroblasts with individual AOs raised hnRNPA1b exon 7b inclusion by approximately 20–40% based upon semiquantitative RT-PCR analysis (Figure 2). As the proposed model for hnRNPA1 exon 7b exclusion involves simultaneous binding of splice repressor to multiple splice silencers, we explored the relative effectiveness of combinations of AOs targeting four different silencing motifs. We combined AO9, which targets the proximal CE1A silencer, with other AOs masking either the CE4 (AO8), CE6 (AO7) and CE9 (AO 4) silencers. Interaction of CE1A and CE4 motifs was demonstrated to facilitate the exclusion of hnRNPA1 exon 7b, [16] and our AO cocktails (9 + 8) may destabilise the hnRNPA1 pre-mRNA loop, promoting inclusion of hnRNPA1 exon 7b and thereby increasing production of the hnRNPa1b isoform.

Seven possible combinations were tested for hnRNPA1b induction (Table 1). These induced 35–40% hnRNPA1b exon 7b inclusion, as assessed at the protein level, and the AOs 9 + 8 + 7 combination was the most effective AO cocktail at inducing hnRNPA1b protein expression (Figure 4). Increased hnRNPA1b expression was associated with inclusion of exon 7 in up to 80% of the SMN2 transcripts (Figure 3), and this effect translated into significantly increased FL-SMN protein expression (Figure 4).

Antisense oligomers with the uncharged morpholino backbone have proved to be promising for clinical use as steric blockers to redirect splicing, as these compounds possesses excellence safety profiles and are not degraded by known nucleolytic enzymes [22,23,24]. Four PMOs, i.e., PMOs 9, 8, 7, 4 were assessed in seven combinations and tested for their splice switching activity in SMA fibroblasts. Treatment of selected combination of PMO strongly increased expression of the hnRNPA1b isoform, but also induced *SMN2* exon 5 skipping without substantial induction in SMN protein expression. We postulated that this effect was caused by disproportionate ratio of hnRNPA1/hnRNPA1b (50–60% hnRNPA1b) and *SMN2* exon 5 skipping. This result prompted us to investigate whether induction of hnRNPA1b may have a global effect on gene expression in SMA fibroblasts. The transcriptome of cells transfected with PMOs9 + 8 + 7 combination was chosen for comparison to that of untreated SMA fibroblasts. Differential Expression analysis indicated decreased expression of survivin (BIRC5), Aurora kinase B (AURKB) and marker of proliferation ki-67 (MKI67) in PMOs9 + 8 + 7 cocktail treated SMA fibroblast. In keeping with this result, cardiomyocytes from the severe mouse model of SMA underwent cell cycle arrest and these three genes were also upregulated in response to increased SMN expression [25]. PMOs9 + 8 + 7 may decrease BIRC5, AURKB and MKI67 through modulating SMN expression through SMN exon 5 skipping. Gene ontology (GO) and gene set enrichment analysis (GSEA) on differentially expressed gene list were enriched for pathways involving cell cycle control, mitosis, nuclear division and chromosome segregation. The top twenty pathways identified by GSEA were statistically significant and nine out of those twenty pathways include the key master regulators, i.e., *FOXM1* or *MYBL2*. RT-PCR demonstrated that treatment with the PMO cocktail reduced *MYBL2* and *FOXM1b* expression in a type I SMA patient cells. MYBL2 and FOXM1, form complex with MuvB, and facilitate cell cycle progression through transcriptional activation of the cell cycle gene homology region (CHR) motif [26]. Cell cycle arrest and senescence is reported to be induced in MYBL2 or FOXM1 deficient cells, [27,28] while overexpression of FOXM1b contributes to the pathogenesis of many cancers i.e., colorectal, [29] nasopharyngeal [30] and renal cancer [31]. hnRNPA1 was reported to antagonise senescence in human lung fibroblasts, [32] and our PMO cocktail mediated switching between two hnRNPA1 isoforms, thereby reducing the expression of key regulators involved in the cell senescence process. 

## 4. Materials and Methods

### 4.1. Culture Conditions of SMA Cell Line

A fibroblast cell line derived from a type I SMA patient (GM03813) was purchased from the Coriell Cell Repository (Camden, NJ, USA). The fibroblasts were maintained in Dulbecco’s modified Eagle medium (DMEM) with high glucose (Invitrogen, Waltham, MA, USA) containing penicillin G (100 units/mL), streptomycin (100 µg/mL) and 10% (*v*/*v*) foetal bovine serum (FBS). Fibroblasts were trypsinised and passaged two to three times per week using 0.25% (*w*/*v*) trypsin-EDTA, and were seeded into either a 24-well plate at a density of 1.5 × 10^4^ cells or a 25 cm^2^ cultured flask at a density of 0.2 × 10^6^ cells for AO transfection.

### 4.2. Antisense Oligonucleotides Design and Transfection into SMA Fibroblasts In Vitro

Four Intronic splice silencers (ISS) identified by Blanchette et al. [16] were chosen for AO targeting. Ten 2′ O-methyl modified AOs on a phosphorothioate backbone were designed to anneal to these four ISSs, as well as an exonic sequence enhancer (ESE) that was predicted by ESE finder 3.0, [33,34], as shown in Figure 1. The nomenclature of the AOs contains the gene name followed by the number of the targeted exon and the coordinates of the AO annealing site relative to either acceptor (A) or donor splice site (D). The AO HBS1LE6A(+114+139) designed to target *HBS1L* was used as a negative/sham control, and had been shown to be inactive at modulating SMN splicing. The AO hSMNE7D(-10-29), designed to target a strong intronic silencer element ISS-N1, is a potent FL-SMN transcript inducer, and was used as a positive control to promote SMN expression. The AO coordinates used in this study are listed in the supplementary Table S1. Each AO was individually transfected into SMA fibroblasts to determine its effects on the splicing pattern of hnRNPA1. Lipofectin reagent (LifeTechnology, Waltham, MA, USA) was used for AO transfection at a ratio of 2:1 Lipofectin to AO, as previously reported [35]. Briefly, lipofectin was mixed with DMEM to a final volume of 100 µL. After ten minutes, the lipofectin mixture was combined with 100 µL of the AO diluted in DMEM. The mixture was then made up to a final volume of 1 mL using antibiotic-free DMEM. At the time of transfection, 500 µL aliquots of this mixture were transferred to each well of a 24-well plate containing SMA fibroblasts and incubated for 48 h.

### 4.3. Transfection of Phosphorodiamidate Morpholino Oligomers in SMA Fibroblasts

Six PMOs (PMOs 4, 7, 8, 9, anti-ISS-N1 and a negative control from Gene Tools) were purchased from Gene Tools LLC (Philomath, OR, USA). PMOs were transfected into the cell by nucleofection using the P2 primary kit (Lonza, Basel, Switzerland). Two microliters of stock PMOs were added into a cuvette, and 250,000 of SMA fibroblasts were resuspended into 18 µL of prewarmed transfection solution, and then added to the cuvette. The CA-137 protocol on the Amaxa 4D machine (Lonza, Basel, Switzerland) was used for nucleofection. Nucleofected fibroblasts were incubated at 37 °C for 3 days prior to harvesting for further analysis.

### 4.4. RNA Extraction and RT-PCR

Total RNA was harvested from transfected Type I SMA fibroblasts 48 h after treatment with AOs using Trizol reagent, according to the manufacturer’s protocol (Invitrogen, Waltham, MA, USA). The RNA solution was then stored at −20 °C until use. Primers used to assess expression of *hnRNPA1*, *SMN* and other transcripts are listed in the supplementary Table S2 [36,37,38,39,40,41,42,43]. Approximately 200 ng of total RNA from each extraction was used as templates for single-step RT-PCR (Invitrogen, Waltham, MA, USA). Amplicons from the hnRNPA1 and SMN RT-PCRs were fractionated on 2% agarose gels and images were captured after staining with ethidium bromide. Product intensity was measured on an ImageQuant LAS 4010 (GE Healthcare Life Sciences, Chicago, IL, USA) and densitometric analysis was determined using image J software [44]. Percentage of *SMN2* exon 7 inclusion is calculated by FL-SMN signal divided by the summation of density values of FL-SMN and SMNΔ7. The percentage of *hnRNPA1* inclusion was determined as the density of exon 7b inclusion divided by the density values of the hnRNPA1 band and the hnRNPA1b band.

### 4.5. RNA Extraction and Transcriptomic Analysis

Total RNA was extracted from type I SMA fibroblasts transfected with PMOs 9 + 8 + 7 or untreated SMA fibroblasts using the RNeasy Mini Kit (Qiagen, Hilden, Germany) according to manufacturer’s protocol. RNA Sequencing (RNA-Seq) was performed by Illumina using 150 bp pair-end with 6 GB of output on the Hiseq. FastQC was used to filter out poor quality reads from FASTQ files [45]. Filtered reads were mapped against human reference genome (GRCh38) using Hisat2 v2.1.0 [46]. Picard Tools was used to remove duplicates [47]. HTseq was employed to generate input for differential expression analysis [48]. DESeq2 v-1.24.0 was used for differential gene expression analysis [49]. gProfiler2 was used for gene ontology analysis, [49,50], while GSEA-P was used for gene set enrichment analysis [51].

### 4.6. Immunoblotting

Three days after transfection, treated SMA fibroblasts were lysed in TRIS glycine buffer at pH 6.8, 10% glycerol with 4% SDS and sonicated with six pulses of 30% amplitude for 30 s on ice using Vibra-cellTM (Sonics and Materials INC., Newtown, CT, USA). Protein concentrations in extracts were measured using a BCA kit (Piercenet). Extracts were centrifuged for 5 min at 10,000× *g* at 4 °C. Sodium dodecyl sulphate (SDS)-PAGE electrophoresis was performed using 10% Tris-glycine gels and run at 100 volts for one hour at 4 °C. After electrophoresis, the gel was removed from the glass plates and the proteins were transferred to FluoroTrans^®^ (Pall, Victoria, Australia) PVDF transfer membranes at 100 volts for 2 h at 4 °C using Omnipage sub blot system transfer system (Cleaver Scientific, Rugby, UK). Two primary antibodies were used: MANSMA1 (Santa Cruz Biotechnology, Dallas, TX, USA), a mouse anti-SMN monoclonal antibody, was applied at a 1:100 dilution for SMN protein detection, whereas the 9H10 antibody (Abcam, Cambridge, UK), a mouse anti-hnRNPA1 monoclonal antibody, was applied at 1:1000 dilution for hnRNPA1 protein detection. A rabbit β-tubulin monoclonal antibody (Abcam, Cambridge, UK) was used as a reference protein loading control. Secondary horseradish peroxidase (HRP)-conjugated antimouse or rabbit IgG antibodies were used at the dilution of 1 in 10,000. Images of the membrane were obtained using ImageQuant LAS 4010. Band intensity was analysed using the ImageJ software [43].

### 4.7. Statistical Analysis

Data are expressed as means ± standard deviation based on three separate experiments and the student T-test was used for statistical analysis.

## 5. Conclusions

In summary, our data confirm the role of hnRNPA1 and its isoforms in FL-SMN transcript expression, and that induction of the hnRNPA1b isoform is readily achievable using an optimised combination of AO sequences. Different levels of hnRNPA1b protein induction regulated exon selection during *SMN2* pre-mRNA splicing. Induction of hnRNPA1 using PMO cocktail in SMA fibroblasts led to reduced expression of the master regulator of cell cycle progression. However, the effective PMO concentration range that can rescue expression of SMN protein appears to be narrow, and optimisation of the PMO cocktail, i.e., different dosing and the chosen AO component in the cocktail, should be evaluated together to refine the appropriate dosage. Further studies that use PMO treatment in animal models are required to determine effective dosage windows. The induction of the hnRNPA1b isoform in SMA mice would be a crucial avenue of study to provide a good indicator of the effectiveness of AO cocktails in the treatment of SMA.

## Figures and Tables

**Table 1:** List of AO combinations targeting the four splice silencing motifs located in introns adjacent to hnRNPA1 exon 7b.

AO Combination	Silencing Motifs Targeted
AOs9 + 8	ISS(CE1A) + ISS(CE4)
AOs9 + 7	ISS(CE1A) + ISS(CE6)
AOs9 + 4	ISS(CE1A) + ISS(CE9)
AOs9 + 8 + 7	ISS(CE1A) + ISS(CE4) + ISS(CE6)
AOs9 + 8 + 4	ISS(CE1A) + ISS(CE4) + ISS(CE9)
AOs9 + 7 + 4	ISS(CE1A) + ISS(CE6) + ISS(CE9)
AOs9 + 8 + 7 + 4	ISS(CE1A) + ISS(CE4) + ISS(CE6) + ISS(CE9)

## Data Availability

RNAseq Data presented in this study are submitted to Sequence Read Archive database (SRA) at Bioproject: PRJNA821645.

## Supplementary Materials

The following supporting information can be downloaded at: https://www.mdpi.com/article/10.3390/ijms23073937/s1.
