# Supplementary material for: Antisense Oligonucleotide Induction of the hnRNPA1b Isoform Affects Pre-mRNA Splicing of SMN2 in SMA Type I Fibroblasts
Source: Int J Mol Sci. 2022 Apr 1;23(7):3937. doi: 10.3390/ijms23073937 (PMC8999010; doi:10.3390/ijms23073937)
Supplement: Supplementary file 1 [file ijms-23-03937-s001.zip › hnRNPA1 paper supp info.pdf]

Supplementary Table S1: List of AO and PMO Sequences used for this study.

AO/ PMO Number	Annealing Coordinates	Sequence 5′ → 3′	Length (bp)
1	hnRNPA1 E7A (+109+133)	CCG CCT CCG TTG TTA TAG CTG TCA T	25
2	hnRNPA1 E7A (+121+145)	CCA AAG CCG CCT CCG CCT CCG TTG T	25
3	hnRNPA1 E7BD (-401-420)	ATT CAG TTG AAT AAT CCA GT	20
4	hnRNPA1 E7BD (-421-440)	TTT CAT TCT CTG GGA AAG GC	20
5	hnRNPA1 E7BD (-411-430)	TGG GAA AGG CAT TCA GTT GA	20
6	hnRNPA1 E7BD (-120-144)	AAG CTA GGA GGA GGT AGC ATA GCA A	25
7	hnRNPA1 E7BD (-145-166)	GGA GTG AGG CGG CCC CAG CTT A	22
8	hnRNPA1 E7BD (-35-59)	AAG CTC TAA AAG GCT AAT CTA GCT G	25
9	hnRNPA1 E7D (-43-62)	TAA CCT ATT CTA AAG ATC CA	20
10	hnRNPA1 E7BD (-167-192)	ACT ATC CAC TTA TCT CTA CTA TTT G	25
Sham AO	HBS 1LE6A (+114+139)	TTC TGA TGC TTG AAT CGT GTG GGG TG	26
Anti-ISS-N1	hSMNE7D (-10-29)	AUU CAC UUU CAU AAU GCU GG	20
PMO Standard Control from Gene Tools		CCTCCTACCTCAGTTACAATTTATA	25

Supplementary Table S2: Primers for the RT-PCR analysis used in this study.

Primer	Sequence (5'--3')	Reference
SMN F	AGGTCTCCTGGAAATAAATCAG	Flynn, LP. 2018
SMN R	TGGTGTCATTTAGTGCTGCTCT	Flynn, LP. 2018
hnRNPA1 F	GAGCTGTCTCCAGAGAAGAT	New design
hnRNPA1 R	CATGTCTTCTTTGTAGCAGCT	New design
hnRNPA2/B1E1F	AAATCGGGCTGAAGCGACTGA	Heartfield, JT. 2002
hnRNPA2/B1E3/4R	CCTCATTACCACACAGTCTGTA	New design
A2B1_3'UTR_A	TTTCAACAGCTGAGGCAAGC	Bonomi, S. 2013
A2B1_3'UTR_B	AGTTACCTGCAGCAAGACACC	Bonomi, S. 2013
hENAHE10/11F	CTTCAACAAGTACACCTGAACC	New design
hENAHE14/15R	AGTTCCTGCCTGATTGCATCAA	New design
hRON2507F	CCTGAATATGTGGTCCGAGACCCCCAG	Ghigna, C. 2005
hRON2991R	CTAGCTGCTTCCTCCGCCACCAGTA	Ghigna, C. 2005
FOXM1a F	GTCTCCACAATTGCCCGAG	Zhang, X. 2017
FOXM1a R	CCAAAATCTCGCAGATCGC	Zhang, X. 2017
FOXM1b F	GGTGTTTAAGCAGCAGAAAC	Zhang, X. 2017
FOXM1b R	GCAGCACCTTGGGGGCAATGC	Zhang, X. 2017
FOXM1c F	CCACTGGACCCAGGGTCTCC	Zhang, X. 2017
FOXM1c R	GCAGCACCTTGGGGGCAATGC	Zhang, X. 2017
FOXM1d F	CAGGTGTTTAAGCAGCAGA	Zhang, X. 2017
FOXM1d R	GGTGATGGGTGTACCAAAAT	Zhang, X. 2017
MYBL2 F1	GGAAGTCTTCTGACCAACTGGC	Karni, R. 2007
MYBL2 R1	GCAGCATGTTTCTGGTGCAGGGG	Karni, R. 2007
MYBL2 F3	AAAACAGTGAGGAGGAAC	Ren, F. 2015
MYBL2 R3	CAGGGAGGTCAAATTTAC	Ren, F. 2015
hGAPDH F	GCAGGGGGGAGCCAAAAGGG	Päth, G. 1997
hGAPDH R	TGCCAGCCCCAGCGTCAAAG	Päth, G. 1997
